# The Impact of Patient Age ≥80 Years on Postoperative Outcomes and Treatment Costs Following Pancreatic Surgery

**DOI:** 10.3390/jcm10040696

**Published:** 2021-02-10

**Authors:** Andreas Andreou, Pauline Aeschbacher, Daniel Candinas, Beat Gloor

**Affiliations:** Department of Visceral Surgery und Medicine, Inselspital, Bern University Hospital, University of Bern, 3010 Bern, Switzerland; andreas.andreou@insel.ch (A.A.); pauline.aeschbacher@insel.ch (P.A.); daniel.candinas@insel.ch (D.C.)

**Keywords:** pancreatic surgery, elderly, octogenarian, mortality, morbidity, cost recovery

## Abstract

As life expectancy is increasing, elderly patients are evaluated more frequently for resection of benign or malignant pancreatic lesions. However, the impact of age on postoperative morbidity, mortality, and treatment costs in octogenarian patients (≥80 years) undergoing major pancreatic surgery needs further investigation. The clinicopathological data of patients who underwent pancreatic surgery between January 2015 and March 2019 in a major hepatopancreatobiliary center in Switzerland were assessed. Postoperative outcomes and hospital costs of octogenarians and younger patients were compared in univariate and multivariate regression analysis. During the study period, 346 patients underwent pancreatic resection. Pancreatoduodenectomy, distal pancreatectomy, total pancreatectomy, and other procedures were performed in 54%, 20%, 13%, and 13% of patients, respectively. The major postoperative morbidity rate and postoperative mortality rate were 25% and 3.5%, respectively. A total of 39 patients (11%) were ≥80 years old, and 307 patients were <80 years old. The majority of octogenarians suffered from ductal adenocarcinoma, whereas among younger patients, other indications for a pancreatic resection were predominant (ductal adenocarcinoma 64% vs. 41%, *p* = 0.006). Age ≥80 was associated with more frequent postoperative medical (pulmonary, cardiovascular) and surgical (high-grade pancreatic fistula, postoperative hemorrhage) complications. Postoperative mortality was significantly higher in octogenarians (15.4% vs. 2%, *p* < 0.0001). This finding may be explained by the higher rate of type C pancreatic fistula (13% vs. 5%), resulting more frequently in postoperative hemorrhage (18% vs. 5%, *p* = 0.002) among patients ≥80 years old. In the multivariate logistic regression analysis, patient age ≥80 years predicted postoperative mortality independently of the tumor entity and surgical technique (*p* = 0.013, OR 6.71, 95% CI [1.5–30.3]). Increased major postoperative morbidity was responsible for lower cost recovery in octogenarians (94% vs. 102%, *p* = 0.046). In conclusion, patient age ≥80 years is associated with increased postoperative medical and surgical morbidity after major pancreatic surgery leading to lower cost recovery and a lower chance for successful resuscitation in patients requiring revisional surgery for postoperative hemorrhage and/or pancreatic fistula. In octogenarian patients suffering from pancreatic tumors, careful selection, and thorough prehabilitation is crucial to achieve the best postoperative and long-term oncologic outcomes.

## 1. Introduction

Pancreatic cancer is currently increasing, as is the aging population worldwide. The ≥80 years old population is expected to grow by a factor of four in the next four decades [1].

Pancreatic surgery has significantly evolved over the past decades. Surgical techniques for the pancreas have also been gradually modified to achieve better functional results [2]. Minimally invasive surgery has been introduced in order to minimize surgical trauma [3]. Successive advances in surgical and perioperative management made pancreatic surgery safer and significantly reduced postoperative mortality and morbidity [4,5]. As a result of the widened technical possibilities and improved postoperative outcomes in pancreatic surgery, the indication for pancreatic resection has been extended to benign lesions, such as intraductal papillary mucinous neoplasm (IPMN) and chronic pancreatitis [2,6,7]. Moreover, diagnostic tools, such as computed tomography (CT) and magnetic-resonance imaging (MRI), have been increasingly used with enhanced quality and are partially responsible for the growing number of newly found pancreatic lesions [8]. For all these reasons, the number of pancreatic resections performed continues to grow.

Additionally, with the increasing aging of the population, more diagnoses of pancreatic pathologies requiring surgery have been identified in the older population, and even more are expected to be diagnosed in the near future [9,10,11]. Innovations in surgical, endoscopic, and anesthesiologic treatment, as well as the establishment of modern neoadjuvant systemic therapies, have enabled more patients with advanced age diagnosed with pancreatic ductal adenocarcinoma (PDAC) to undergo surgery. Thus, elderly patients previously considered as unresectable, due to relevant comorbidities or advanced disease are currently more frequently selected as eligible candidates for curative resection [12]. 

Previous studies investigating age-associated postoperative morbidity and mortality in pancreatic surgery have demonstrated conflicting results. Some single-center studies did not find age as a negative prognostic factor for postoperative outcomes [8,9,13]. On the other hand, population-based studies found a negative association between older age and postoperative morbidity and mortality following pancreatic surgery [11,14,15].

Patient age has been associated with higher postoperative complication rates, and surgical morbidity has been related to increased treatment costs in recent studies focusing on pancreatic resections [16,17]. Political and hospital administration, as well as health insurance companies, lay great importance on this matter and demand the best possible cost-effectiveness.

As more elderly patients are diagnosed with pancreatic pathologies requiring resection, surgeons need to evaluate operability, taking into account multiple factors, such as concomitant comorbidities, expected perioperative morbidity and mortality, and estimated long-term prognosis in order to optimize the selection of appropriate surgical candidates. We hypothesized that postoperative morbidity and mortality following pancreatic surgery is higher in patients ≥80 years old as compared to younger patients. Consequently, treatment costs are also expected to be higher in the elderly population. Therefore, the aim of this study was to compare postoperative morbidity, mortality, and treatment cost recovery in patients younger and older than80 years undergoing pancreatic resection in a major hepatopancreatobiliary center in Switzerland.

## 2. Material and Methods

### 2.1. Patient Inclusion Criteria

This study was approved by the Cantonal Ethical Committee of Bern (2019-01171). The anonymity of the patients in this study was preserved at any time during data acquisition and analysis. In this retrospective study [18], all consecutive patients who underwent pancreatic resection for benign and malignant pancreatic lesions between January 2015 and March 2019 at the Department of Visceral Surgery und Medicine, Inselspital, Bern University Hospital were included. Patients < 18 years old were excluded from the analysis.

### 2.2. Preoperative Management

The standard preoperative patient evaluation included medical history, physical examination, serum laboratory tests, imaging studies, and an anesthesia evaluation. In the case of malignant tumor, the location and extent of tumor burden, as well as the presence of lymph node or distant metastases, were determined by cross-sectional imaging, such as contrast-enhanced CT and/or MRI.

In the case of obstructive jaundice, some patients underwent bile decompression using bile duct stents during endoscopic retrograde cholangiopancreatography, endoscopic hepaticogastrostomy, or percutanous transhepatic drainage. The decision to proceed with a bile decompression was individually made according to the duration and extent of cholestasis and the general condition of the patient.

All patients presenting with a malignant tumor were preoperatively discussed at our interdisciplinary tumor board, including pancreatic surgeons, medical oncologists, radiation therapists, and specialized radiologists. Tumor resectability was assessed, and an individualized course of treatment was established for each patient. In patients with borderline resectable or locally-advanced adenocarcinoma, preoperative chemotherapy was recommended, as also described by others [19].

Surgical indication for patients ≥80 years old with pancreatic pathology did not differ from that of younger patients. Medical preconditions and nutritional status [20] were thoroughly evaluated in all patients to identify patients with increased risk for postoperative medical and surgical complications. In the case of relevant comorbidities or nutritional deficiency, preoperative interventions and nutritional prehabilitation were performed to optimize the patients’ status prior to surgery. Extensive preoperative counseling was performed, especially with octogenarians, in order to inform patients in detail regarding potential postoperative complications and complicated postoperative clinical course.

### 2.3. Surgical Procedure

Pancreatic resections included pancreatoduodenectomy (PD), distal pancreatectomy, total pancreatectomy, duodenum-preserving pancreatic head resection, and segmental pancreatic resection. Necrosectomies for pancreatitis were excluded. Resections were either performed using a conventional open technique or a laparoscopic-assisted procedure beginning in 2017. Following an upper transverse laparotomy or diagnostic laparoscopy, previously undiagnosed tumor spread into the peritoneum or the liver was ruled out. Classic Kausch-Whipple or pylorus-preserving PD was performed to remove the pancreatic head as previously described [21]. Reconstruction was performed by pancreatojejunostomy using a dissected jejunal loop, end-to-side hepaticojejunostomy, and gastrojejunostomy to re-establish the gastrointestinal passage. Perianastomotic drains were intraoperatively placed to monitor pancreatic fistula, anastomotic leak, and postoperative hemorrhage. In the case of tumor involvement of the portal vein/superior mesenteric vein or superior mesenteric artery, vessel resection, and reconstruction either with anastomosis or graft interposition were performed. Irreversible electroporation (IRE) was used for margin accentuation in patients with a borderline resectable disease to the hepatic artery, celiac trunk, or superior mesenteric artery [22,23].

### 2.4. Postoperative Management

After surgery, all patients were monitored for postoperative complications. Remarkable discharge from perianastomotic drains or persistently increased bilirubin, or lipase levels in drained fluids were indicators for biliary leak and pancreatic fistula, respectively. The criteria of the International Study Group on Pancreatic Fistula (ISGPS) were used for classifying postoperative pancreatic fistulas (POPF) in three severity grades (biochemical leak or A, B, and C), and patients were treated accordingly [24]. Further complications were documented, such as postoperative hemorrhage (defined as hemodynamic- and hemoglobin-relevant bleeding [25]), wound infection (defined as surgical site infection [26]), pulmonary (including pneumonia and pleural effusions), cardiovascular (including hemodynamic instability, cardiac insufficiency, myocardial infarction), and renal complications (including renal failure) [27].

Postoperative morbidity was defined as any complication within 90 days after the surgical procedure and was classified according to a standardized classification [28]. Postoperative complications ≥ grade 3 were defined as major morbidity. The postoperative mortality rate was defined as the fraction of patients suffering any complication within 90 days following surgery leading to death.

Postoperative chemotherapy and radiotherapy were administered in patients based on recommendations of the interdisciplinary tumor board.

### 2.5. Statistical Analysis

The primary endpoint of this study was the comparison between patients ≥80 years old vs. patients <80 years regarding postoperative 90-day mortality. Secondary endpoints were the comparison of the two groups in regard to postoperative 90-day morbidity, cost recovery (CR), defined as the ratio treatment costs/proceeds in percent according to the Swiss diagnosis-related groups (DRG) reimbursement system, and overall-survival (OS). These objectives were investigated in the entire cohort of patients who underwent pancreatic resection during the study period, as well as in subgroups of patients undergoing PD, and patients undergoing resection for PDAC.

Quantitative and qualitative variables were expressed as medians (range) and frequencies. The Chi-square or Fisher’s exact test, and the Mann-Whitney *U* test were used to compare categorical and continuous variables, as appropriate. 

To identify factors associated with postoperative mortality after pancreatic resection, clinicopathological variables were analyzed in univariate analysis. In the subsequent multivariate analysis, all factors with *p* value < 0.05 in univariate analysis were entered in a logistic regression model to identify independent predictors for postoperative mortality.

Using the Kaplan-Meier method, OS was calculated for patients with PDAC from the date of surgical procedure to the date of death or last follow-up. Log-rank test was used to compare OS between patients ≥80 years old vs. patients <80 years old.

*p* values < 0.05 were considered statistically significant. For statistical analysis, SPSS software package, version 25, by IBM (Armonk, NY, USA) was used.

## 3. Results

### 3.1. Patient Characteristics and Postoperative Outcomes in the Entire Cohort

From January 2015 to March 2019, 346 patients underwent pancreatic resection, 307 patients were <80 years old, and 39 patients (11%) were ≥80 years old. In-depth information on the entire cohort and the treatment is given in Table 1. Pancreatic pathologies, including PDAC, periampullary cancers, cystic lesions, neuroendocrine tumors, and chronic pancreatitis, were not significantly differently distributed between patients ≥80 years old and patients <80 years old (*p* = 0.127). The major postoperative morbidity rate and 90-day postoperative mortality rate for the entire cohort were 25% and 3.5%, respectively. POPF type C, postoperative hemorrhage, pulmonary and cardiovascular complications were significantly higher among patients ≥80 years old. Postoperative mortality was also significantly higher among patients ≥80 years old (15% vs. 2%, *p* < 0.0001). Cost recovery calculation revealed unfavorable outcomes for the older patients (94% (46–172) vs. 102% (38–233), *p* = 0.046). The median hospital costs in our cohort were 38’185 (15’763–176’318) CHF.

### 3.2. Comparison of Patients Undergoing Pancreaticoduodenectomy

In Table 2, data is focusing on the PD, as it is the most challenging of pancreatic resections. PD was performed in 185 patients during the study period, and 26 patients (14%) were ≥80 years old. Postoperative mortality was higher in the ≥80 years old group (11.5% vs. 1.3%, *p* = 0.003). There were also more POPF type C, postoperative hemorrhage, and cardiovascular complications in the ≥80 years old group.

### 3.3. Comparison of Patients Undergoing Surgery for Pancreatic Ductal Adenocarcinoma 

In Table 3, the focus is on patients suffering from PDAC. This group consisted of 150 patients, and 25 patients (17%) were ≥80 years old. Postoperative mortality was higher among patients ≥80 years old (*n* = 5 vs. *n* = 3, *p* < 0.0001). TNM stage and resection margins status were comparable in the two groups. However, the proportion of patients ≥80 years old who received adjuvant chemotherapy was significantly lower in comparison to the <80 years cohort (48% vs. 79%, *p* = 0.001).

### 3.4. Factors Associated with Postoperative Mortality

Table 4 summarizes the results of the univariate and multivariate analysis of factors associated with 90-day postoperative mortality. Multivariate logistic regression analysis showed that patient age ≥80 years was associated with postoperative mortality, independently of the tumor entity and surgical technique (*p* = 0.013, OR 6.71, 95% confidence interval (CI) (1.5–30.3)). Other predictive factors for postoperative mortality were postoperative hemorrhage (*p* = 0.001, OR 12.21 95% CI (2.7–55.9)), and renal complications (*p* = 0.001, OR 15.80, 95% CI (3.1–81.4)). Table 5 provides detailed information on the six patients ≥80 years old suffering postoperative 90-day mortality following pancreatic surgery, including postoperative complications and cause of death. Among the six octogenarians who died after pancreatic surgery, three patients suffered POPF type C, and four patients required re-operation because of postoperative hemorrhage. In this group of postoperative mortality, three patients underwent a Whipple procedure, but only two of them suffered POPF. One patient had a soft pancreatic texture, and the other one hard pancreatic texture. Additional to the surgical complications, the cause of death in three patients included respiratory failure (due to aspiration pneumonia), cardiac arrest (due to myocardial infarction), and liver failure (due to thrombosis of the common hepatic artery), respectively (Table 5).

### 3.5. Long-Term Survival of Patients with PDAC

**Figure 1:**
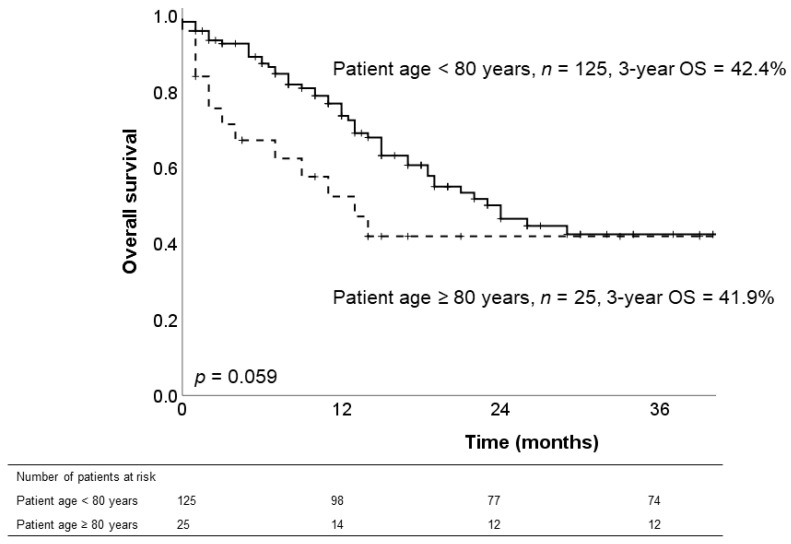
Comparison of overall survival (OS) in patients with pancreatic ductal adenocarcinoma (*n* = 150) according to patient age (<80 years vs. ≥80 years).

Among patients with PDAC, patient age was not associated with oncologic outcome using a cut-off value of 80 years. Three-year overall survival rates were comparable between patients <80 years old and patients ≥80 years old (42.4% vs. 41.9%, *p* = 0.059, Figure 1). The median survival was 24 (18–30) months for patients <80 years old vs. 13 (6–20) months for patients ≥80 years old (*p* = 0.059).

We analyzed the impact of sex on OS in the subgroups of patients <80 years old and patients ≥80 years old. We found no statistically significant survival difference between female and male patients, both in patients <80 years old (female, *n* = 56, 3-year OS 45% vs. male, *n* = 68, 3-year OS 40.8%, *p* = 0.638) and patients ≥80 years old (female, *n* = 14, 3-year OS 51.6% vs. male, *n* = 11, 3-year OS 31.8%, *p* = 0.571), even though OS was higher for female patients in both groups.

## 4. Discussion

In an unselected group of 346 consecutive patients, our study examined the impact of patient age on postoperative mortality and morbidity after pancreatic resective surgery. Importantly, we focused not only on the entire cohort, but also on the group of patients undergoing PD and on those suffering from PDAC.

Patient age ≥80 years was significantly associated with a higher rate of both surgical and medical complications in our study. Additionally, the postoperative mortality rate was higher in our cohort of patients ≥80 years old. These results are comparable to those of previous studies, including a large study from Germany, investigating the results of 1705 pancreatic operations, including 76 (4.5%) patients ≥80 years old [8]. A recent international multicentric study with 3624 elderly patients from Belgium, the Netherlands, and Norway showed postoperative mortality rates beyond 10% for patients ≥80 years old, underlining the importance of investigating the role of patient age on outcomes following pancreatic surgery [15]. The aim of this study was not to determine a cut-off age for pancreatic surgery, but to underline the importance of correct identification of the candidates who will benefit from surgery the most, despite advanced age. Elderly patients are more fragile and have fewer capacities to overcome major postoperative complications [29].

A higher rate of medical complications, such as pneumonia and cardiovascular adverse events, has been found in the cohort of patients ≥80 years old in our study. Elderly patients presented more frequently with preoperative cardiovascular and pulmonary comorbidities, which may have deteriorated postoperatively. The association between relevant comorbidities and increased postoperative complications has been previously discussed [30]. In this regard, frailty among elderly patients is known as a state of decreased capacity to cope with stress factors in order to restore homeostasis [1]. However, frailty is an independent factor that does not always correlate with age and may also be reversible [31]. Our results, indicating that the elderly population is more vulnerable to postoperative complications with a lower capacity to recover, underline the need for precise preoperative assessment of medical preconditions in order to improve patient selection and reduce the risk for selected patients. Patient-level factors contributed the most to the increased risk of mortality after PD in a recent US study [32]. Additionally, interventions must be undertaken prior to surgery to improve nutritional status [20], and the medical comorbidities, when possible. It is important to mention that we have conducted even more extensive conversations with the older patients, mostly together with their families, in order to find out how their physical capacity in daily life was and how much treatment they wished to undergo. Based on the results of our study showing that patient age is a prognostic factor for adverse events after surgery, elderly patients and their close family members were made aware of this risk during preoperative counseling. They were explicitly informed of the possible need for prolonged intensive care, respiratory, cardiovascular, and renal support, in the case of a complication. If, during the preoperative counseling, life-support measures were denied by the patient in the case of a postoperative complication, the indication for major resection was restricted.

ASA status has been identified as a useful tool for preoperative assessment [33]. We showed that the group of patients ≥80 years old had mostly higher ASA scores, however ASA status of 4 was not associated with higher postoperative mortality in our study. Nevertheless, this result may be associated with the low number of ASA 4 patients, and octogenarians with an ASA status 4 are not considered as surgical candidates in our practice anymore.

Patients ≥80 years old suffered more frequently from POPF type C with subsequent hemorrhage [34]. Increased patient age is only one factor among others, such as BMI, visceral fat thickness, pancreatic duct size, and pancreas tissue texture that have been previously shown to be associated with POPF [35,36,37]. Thus, postoperative hemorrhage, due to vessel erosion, may have been responsible for the increased postoperative mortality in the octogenarian cohort, additionally to the increased preoperative risk factors. Patient age ≥80 years was associated with postoperative mortality in the entire cohort, as well as in the subset of patients undergoing pancreaticoduodenectomy, and patients with PDAC.

Postoperative mortality among octogenarians was even higher in patients with PDAC compared to other indications for pancreatic surgery. This difference may be explained by the fact that oncological patients often present with more severe malnutrition and have less capacity to recover [38]. Moreover, even though not statistically significant, there was a trend to more total pancreatectomies among octogenarians. This is in concordance with previous studies as total pancreatectomy is often favored over a partial pancreaticoduodenectomy in high-risk patients with more advanced disease [39]. Nevertheless, patient age was associated with postoperative mortality independently of the tumor entity and surgical technique in the multivariate analysis. However, the large confidence interval (1.5–30.3) for postoperative mortality in the multivariate analysis indicates the relatively small number of patients involved.

Cost recovery following pancreatic resection was worse for patients ≥80 years old in the entire cohort, as well as in patients with PDAC and patients undergoing PD. Higher surgical (POPF, hemorrhage) and medical (pulmonary, cardiovascular) postoperative complications in this cohort have contributed to higher treatment costs, and thus, worse financial balance. Previous studies confirmed that higher costs are directly related to the presence of severe complications and longer hospital stay [17]. Grade III and IV complications cause a doubling of the costs compared to an uneventful postoperative course [17]. Nevertheless, financial criteria and potentially worse cost recovery after surgery are currently not taken into consideration, when evaluating octogenarians or any other patient cohort for pancreatic resection or other oncologic treatment. 

In our study, we have observed that the older population was less likely to receive adjuvant chemotherapy. Postoperative complications (especially pancreatic fistula and postoperative hemorrhage), but also higher age, and lower center volume were predictive factors for not receiving adjuvant chemotherapy after pancreatic resection in a recent Dutch multicentric study [40]. In our cohort, this result can be explained by higher rates of POPF and postoperative hemorrhage among patients ≥80 years old. Additionally, a large proportion of our patients undergoes systemic treatment in peripheral hospitals, where older patients are often treated less aggressively, and higher dropout rates during adjuvant chemotherapy are observed [41]. In our center, we have been increasingly recommending neoadjuvant chemotherapy for 3–6 months for patients with borderline resectable or locally-advanced PDAC in the most recent years. Among 150 patients with PDAC, only 13 patients <80 years were administered preoperative chemotherapy in our study. Due to our relatively strict selection criteria for pancreatic surgery among elderly patients, patients ≥80 years old with such extended tumor burden requiring long preoperative chemotherapy and complex surgery were mostly not appropriate surgical candidates. Additionally, elderly patients ≥80 years old often did not qualify for the preferred regimen with FOLFIRINOX, which has a relevant toxicity profile, as indicated by the study from Conroy et al., which included only patients <80 years in the FOLFIRINOX arm [42].

Our survival analysis for patients with PDAC showed that patient age was not significantly associated with oncologic outcome. Although, mortality has been more frequently observed among patients ≥80 years old in the first two years following oncologic resection, survival did not differ between the two groups on the long-term and 3-year OS was identical (<80 years: 42.4% vs. ≥80 years: 41.9%, *p* = 0.059). This result indicates that prolonged survival is possible for well-selected patients ≥80 years old, who undergo oncologic resection with an uneventful postoperative course, enhanced recovery, and fast return to normal function.

Finally, our study has several limitations. It is a retrospective analysis of an inhomogeneous population of patients, including benign and malignant pancreatic lesions. However, it represents the standard case profile of a high-volume center in Switzerland, reflecting our experience with an octogenarian population. We have performed analysis using the cut-off age of 80 years, acknowledging that results may slightly vary by choosing another value. However, our aim was not to provide evidence on whether to operate on patients ≥80 years old or not, but rather to indicate the challenges older and multimorbid patients requiring pancreatic surgery provide and to discuss ways to improve outcomes in this cohort.

## 5. Conclusions

Patient age of ≥80 years was associated with a higher risk for medical and surgical postoperative complications and mortality following pancreatic surgery. Reduced capacity for successful resuscitation and postoperative recovery when requiring revision surgery among the elderly patient cohort has been identified. Surgical treatment of octogenarians was also related to a lower cost recovery rate. Nevertheless, pancreatic surgery remains the only curative-intended approach for pancreatic cancer, and therefore, careful patient selection is essential to achieve favorable outcomes.

## Figures and Tables

**Table 1:** Comparison of clinicopathological characteristics of 346 patients undergoing pancreatic surgery according to patient age.

Variable	<80 Years (*n* = 307)	≥80 Years (*n* = 39)	All Patients (*n* = 346)	*p* Value
Gender, *n* (%)				0.106
Female	139 (45)	23 (59)	162 (47)	
Male	168 (55)	16 (41)	184 (53)	
cost recovery, %, median (range)	102 (38–233)	94 (46–172)	101 (38–233)	0.046
cardiovascular disease, *n* (%)	157 (51)	33 (85)	190 (55)	<0.0001
Kidney disease, *n* (%)	41 (13)	13 (33)	53 (15)	0.001
Pulmonary disease, *n* (%)	71 (23)	8 (21)	79 (23)	0.714
Infectious disease, *n* (%)	11 (4)	1 (3)	12 (4)	0.743
Liver disease, *n* (%)	43 (14)	7 (18)	50 (15)	0.510
Neurologic disease, *n* (%)	49 (16)	6 (15)	55 (16)	0.926
Diabetes, *n* (%)	73 (24)	11 (28)	84 (24)	0.544
Genetical alteration, *n* (%)	9 (3)	0 (0)	9 (3)	0.279
BMI, kg/m^2^, median (range)	24 (16–46)	24 (16–31)	24 (16–46)	0.167
BMI > 30, kg/m^2^, *n* (%)	48 (16)	4 (19)	52 (15)	0.376
Smoking, *n* (%)	131 (43)	3 (8)	134 (39)	<0.0001
Alcohol consumption, *n* (%)	106 (35)	6 (15)	112 (32)	0.016
ASA status, *n* (%)				0.009
1	7 (2)	0 (0)	7 (2)	
2	98 (32)	5 (13)	103 (30)	
3	190 (62)	29 (74)	219 (63)	
4	12 (4)	5 (13)	17 (5)	
Resection type, *n* (%)				0.122
Pancreatoduodenectomy	159 (52)	26 (67)	185 (54)	
Distal pancreatectomy	63 (21)	6 (15)	69 (20)	
Total pancreatectomy	38 (12)	7 (18)	45 (13)	
Enucleation	3 (1)	0 (0)	3 (1)	
Duodenal-preserving resection	24 (8)	0 (0)	24 (7)	
Segmental pancreatic resection	20 (6)	0 (0)	20 (5)	
Operating time, h, median (range)	5.0 (1–10)	5.75 (3–9)	5.2 (1–10)	0.014
Vascular reconstruction, *n* (%)				0.638
none	240 (78)	28 (72)	268 (78)	
venous	56 (19)	10 (26)	66 (19)	
arterial	7 (2)	1 (2)	8 (2)	
combined	4 (1)	0 (0)	4 (1)	
Histologic type, *n* (%)				0.006
Adenocarcinoma	125 (41)	25 (64)	150 (43)	
other	182 (59)	14 (36)	196 (57)	
Pancreatic pathologies, *n* (%)				0.127
PDAC	125 (41)	25 (64)	150 (43)	
periampullary malignancies	46 (15)	6 (16)	52 (15)	
cystic lesions	64 (21)	5 (13)	69 (20)	
neuroendocrine tumors	28 (9)	2(5)	30 (9)	
chronic pancreatitis	44 (14)	1 (2)	45 (13)	
Length of ICU stay, days, median (range)	1 (0–37)	1 (1–19)	1 (0–37)	0.065
Length of hospital stay, days, median (range)	13 (2–87)	13 (3–66)	13 (2–87)	0.372
Readmission within 90 days, *n* (%)	53 (17)	6 (17)	59 (17)	0.908
Postoperative morbidity, *n* (%)	185 (60)	25 (64)	210 (61)	0.664
Major postoperative morbidity, *n* (%)	73 (24)	12 (31)	85 (25)	0.339
Postoperative mortality, *n* (%)	6 (2.0)	6 (15.4)	12 (3.5)	<0.0001
POPF, *n* (%)				0.035
None	221 (72)	31 (79)	252 (73)	
Type A	43 (14)	3 (8)	46 (13)	
Type B	9 (28)	0 (0)	28 (8)	
Type C	15 (5)	5 (13)	20 (6)	
Re-operation, *n* (%)	42 (14)	9 (23)	51 (15)	0.119
Postoperative hemorrhage, *n* (%)	15 (5)	7 (18)	22 (6)	0.002
Wound infection, *n* (%)	60 (20)	8 (21)	68 (20)	0.886
Pulmonary complication, *n* (%)	34 (11)	10 (26)	44 (13)	0.010
Cardiovascular complication, *n* (%)	15 (5)	8 (21)	23 (7)	<0.0001
Renal complication, *n* (%)	15 (5)	3 (8)	18 (5)	0.457

BMI, body mass index; ASA, American Society of Anesthesiologists; POPF, postoperative pancreatic fistula; ICU, intensive care unit; PDAC, pancreatic ductal adenocarcinoma.

**Table 2:** Comparison of clinicopathological characteristics of 185 patients undergoing pancreatoduodenectomy according to patient age.

Variable	<80 Years (*n* = 159)	≥80 Years (*n* = 26)	All Patients (*n* = 185)	*p* Value
Gender, *n* (%)				0.032
Female	74 (47)	18 (69)	92 (50)	
Male	85 (53)	8 (31)	93 (50)	
Cost recovery, %, median (range)	105 (42–233)	91 (46–172)	102 (42–233)	0.045
Heart disease, *n* (%)	82 (52)	23 (89)	106 (57)	<0.0001
Kidney disease, *n* (%)	21 (13)	8 (31)	29 (16)	0.022
Smoking, *n* (%)	58 (37)	1 (4)	59 (32)	0.001
Alcohol consumption, *n* (%)	54 (34)	2 (8)	56 (30)	0.007
ASA status, *n* (%)				0.123
1	1 (1)	0 (0)	1 (1)	
2	49 (31)	3 (12)	52 (28)	
3	102 (64)	20 (77)	122 (66)	
4	7 (4)	3 (11)	10 (5)	
Diameter pancreatic duct, mm, median (range)	4 (1–15)	4 (3–8)	4 (1–15)	0.080
Pancreatic stent intraoperative, *n* (%)	37 (24)	1 (4)	28 (21)	0.021
Histologic type, *n* (%)				0.603
Adenocarcinoma	83 (52)	15 (58)	98 (53)	
other	76 (48)	11 (42)	87 (47)	
Length of ICU stay, days, median (range)	1 (0–26)	1 (1–11)	1 (0–26)	0.190
Length of hospital stay, days, median (range)	13 (7–67)	13 (3–48)	13 (3–67)	0.973
Readmission within 90 days, *n* (%)	26 (17)	5 (20)	31 (17)	0.661
Postoperative morbidity, *n* (%)	86 (54)	19 (73)	105 (57)	0.07
Major postoperative morbidity, *n* (%)	36 (23)	9 (35)	45 (24)	0.187
Postoperative mortality, *n* (%)	2 (1.3)	3 (11.5)	5 (2.7)	0.003
POPF, *n* (%)				0.452
None	123 (77)	20 (77)	143 (77)	
Type A	12 (8)	2 (8)	14 (8)	
Type B	10 (6)	0 (0)	10 (5)	
Type C	14 (9)	4 (15)	18 (10)	
Re-operation, n (%)	24 (15)	7 (27)	31 (17)	0.134
Salvage pancreatectomy, *n* (%)	13 (8)	4 (15)	17 (9)	0.432
Bile leak, *n* (%)	4 (3)	0 (0)	4 (2)	0.414
Gastrointestinal leak, *n* (%)	2 (1)	0 (0)	2 (1)	0.565
Postoperative hemorrhage, *n* (%)	7 (4)	5 (19)	12 (7)	0.004
Wound infection, *n* (%)	34 (21)	7 (27)	41 (22)	0.528
Pulmonary complication, *n* (%)	20 (13)	6 (23)	26 (14)	0.153
Cardiovascular complication, *n* (%)	6 (4)	6 (23)	12 (7)	<0.0001
Renal complication, *n* (%)	4 (3)	2 (8)	6 (3)	0.167

BMI, body mass index; ASA, American Society of Anesthesiologists; POPF, postoperative pancreatic fistula; ICU, intensive care unit.

**Table 3:** Comparison of clinicopathological characteristics of 150 patients undergoing resection for pancreatic ductal adenocarcinoma according to patient age.

Variable	<80 Years *(n* = 125)	≥80 Years (*n* = 25)	All Patients (*n* = 150)	*p* Value
Gender, *n* (%)				0.306
Female	56 (45)	14 (56)	70 (47)	
Male	69 (55)	11 (44)	80 (53)	
Cost recovery, %, median (range)	103 (47–233)	90 (46–172)	100 (46–233)	0.021
Heart disease, *n* (%)	72 (58)	21 (84)	93 (62)	0.13
Kidney disease, *n* (%)	18 (14)	9 (36)	27 (18)	0.010
Smoking, *n* (%)	48 (38)	3 (12)	51 (34)	0.011
Alcohol consumption, *n* (%)	42 (34)	5 (20)	47 (31)	0.181
ASA status, *n* (%)				0.037
1	2 (2)	0 (0)	2 (1)	
2	33 (26)	1 (4)	34 (23)	
3	80 (64)	19 (76)	99 (66)	
4	10 (8)	5 (20)	15 (10)	
CA 19-9 preoperative, kU/L, median (range)	360 (2–63690)	311 (4–11184)	343 (2–63690)	0.692
IRE, *n* (%)	20 (16)	2 (8)	22 (15)	0.302
Operating time, h, median (range)	6.0 (2–9)	6.4 (4–9)	6.0 (2–9)	0.484
T stage, *n* (%)				0.065
T1	5 (4)	0 (0)	5 (3)	
T2	45 (36)	16 (64)	61 (41)	
T3	74 (59)	9 (36)	83 (55)	
T4	1 (1)	0 (0)	1 (1)	
N stage, *n* (%)				0.221
N0	22 (18)	8 (32)	30 (20)	
N1	74 (59)	11 (44)	85 (57)	
N3	29 (23)	6 (24)	35 (23)	
Lymph node ratio, median (range)	3 (0–37)/ 28 (6–107)	2 (0–10)/ 23 (19–62)	2 (0–37)/ 28 (6–107)	0.169
Lymphagiosis carcinomatosa, *n* (%)	25 (20)	32 (8)	33 (22)	0.186
Venous invasion, *n* (%)	16 (13)	7 (28)	23 (15)	0.054
Perineural invasion, *n* (%)	6 (5)	2 (8)	8 (5)	0.516
Tumor differentiation, *n* (%)				0.484
G1	18 (14)	3 (12)	21 (14)	
G2	50 (40)	14 (56)	64 (43)	
G3	55 (44)	8 (32)	63 (42)	
G4	2 (2)	0 (0)	2 (1)	
Tumor margins, *n* (%)				1.0
R1	40 (32)	8 (32)	48 (32)	
R0	85 (68)	17 (68)	102 (68)	
Length of ICU stay, days, median (range)	1 (0–37)	1 (1–19)	1 (0–37)	0.269
Length of hospital stay, days, median (range)	13 (2–70)	12 (3–66)	13 (2–70)	0.667
Readmission within 90 days, *n* (%)	22 (18)	3 (13)	25 (17)	0.571
Postoperative morbidity, *n* (%)	70 (56)	15 (60)	86 (57)	0.713
Major postoperative morbidity, *n* (%)	25 (20)	8 (32)	33 (22)	0.186
Postoperative mortality, *n* (%)	3 (2.4)	5 (20)	8 (5.3)	<0.0001
POPF, *n* (%)				0.077
None	104 (83)	21 (84)	125 (83)	
Type A	10 (8)	1 (4)	11 (7)	
Type B	8 (7)	0 (0)	8 (6)	
Type C	3 (2)	3 (12)	6 (4)	
Re-operation, *n* (%)	17 (13)	7 (28)	24 (16)	0.073
Postoperative hemorrhage, *n* (%)	6 (5)	6 (24)	12 (8)	0.001
Wound infection, *n* (%)	30 (24)	4 (16)	34 (23)	0.383
Pulmonary complication, *n* (%)	16 (13)	6 (24)	22 (15)	0.148
Cardiovascular complication, *n* (%)	7 (6)	5 (20)	12 (8)	0.015
Renal complication, *n* (%)	9 (7)	2 (8)	11 (7)	0.889
Neoadjuvant chemotherapy, *n* (%)	13 (10)	0 (0)	13 (9)	0.092
Adjuvant chemotherapy, *n* (%)	99 (79)	12 (48)	111 (74)	0.001
Adjuvant radiotherapy, *n* (%)	1 (1)	0 (0)	1 (1)	0.654

BMI, body mass index; ASA, American Society of Anesthesiologists; IRE, Irreversible electroporation; POPF, postoperative pancreatic fistula; ICU, intensive care unit.

**Table 4:** Analysis of factors associated with postoperative mortality in 346 patients undergoing pancreatic surgery.

Variable	Postoperative Mortality (*n* = 12)	No Postoperative Mortality (*n* = 334)	All Patients (*n* = 346)	*p* Value	MV *p* Value, OR (CI)
Male, *n* (%)	7 (58)	177 (53)	184 (53)	0.716	
Age ≥80 years, *n* (%)	6 (50)	33 (10)	39 (11)	<0.0001	0.013, 6.71 (1.5–30.3)
Heart disease, *n* (%)	8 (67)	182 (55)	190 (55)	0.411	
Kidney disease, *n* (%)	2 (17)	52 (16)	54 (16)	0.918	
Pulmonary disease, *n* (%)	5 (42)	74 (22)	79 (23)	0.114	
Alcohol consumption, *n* (%)	2 (17)	110 (33)	112 (32)	0.237	
ASA status 4, *n* (%)	1 (8)	16 (5)	7 (6)	0.577	
Resection type, *n* (%)				0.295	
Pancreatoduodenectomy	5 (42)	180 (54)	185 (53)		
Distal pancreatectomy	3 (25)	66 (20)	69 (20)		
Total pancreatectomy	4 (33)	41 (12)	45 (13)		
Enucleation	0 (0)	3 (1)	3 (1)		
Duodenal-preserving resection	0 (0)	24 (7)	24 (7)		
Segmental pancreatic resection	0 (0)	20 (6)	20 (6)		
Histologic type, *n* (%)				0.097	
Adenocarcinoma	8 (67)	142 (43)	150 (43)		
other	4 (33)	192 (56)	196 (57)		
Vascular reconstruction, *n* (%)				0.001	NS
none	4 (34)	264 (79)	268 (78)		
venous	6 (50)	60 (18)	66 (19)		
arterial	1 (8)	7 (2)	8 (2)		
combined	1 (8)	3 (1)	4 (1)		
POPF, *n* (%)				0.036	NS
None	7 (59)	245 (73)	252 (73)		
Type A	1 (8)	45 (14)	46 (13)		
Type B	1 (8)	27 (8)	28 (8)		
Type C	3 (25)	17 (5)	20 (6)		
Postoperative hemorrhage, *n* (%)	6 (50)	16 (5)	22 (6)	<0.0001	0.001, 12.21 (2.7–55.9)
Wound infection, *n* (%)	3 (25)	65 (20)	68 (20)	0.635	
Pulmonary complication, *n* (%)	6 (50)	38 (11)	44 (13)	<0.0001	NS
Cardiovascular complication, *n* (%)	5 (42)	18 (5)	23 (7)	<0.0001	NS
Renal complication, *n* (%)	6 (50)	12 (4)	18 (5)	<0.0001	0.001, 15.80 (3.1–81.4)

BMI, body mass index; ASA, American Society of Anesthesiologists; MV, multivariate logistic regression analysis; OR, odds ratio; CI, confidence interval; NS, not significant; POPF, postoperative pancreatic fistula.

**Table 5:** Clinicopathological characteristics of the six patients ≥80 years old suffering postoperative 90-day mortality following pancreatic surgery.

Variable	Patient 1	Patient 2	Patient 3	Patient 4	Patient 5	Patient 6
Gender	Male	Male	Female	Male	Female	Female
Age	81	85	80	82	81	84
Comorbidities	no	cardiac	cardiac	cardiac	renal	cardiac
ASA status	2	3	3	3	3	3
Type of resection	Distal pancreatectomy	Distal pancreatectomy	Whipple procedure	Whipple procedure	Whipple procedure	total pancreatectomy
Pancreatic anastomosis	-	-	pancreato-jejunostomy	pancreato-jejunostomy	pancreato-jejunostomy	-
Pancreas texture	soft	soft	hard	soft	hard	soft
Pancreatic duct diameter (mm)	-	-	8	3	4	-
POPF	type A	type C	no	type C	type C	no
Postoperative hemorrhage	no	yes	yes	yes	no	yes
Re-operation	no	yes	yes	yes	yes	yes
Other postoperative complications	myocardial infarction	pneumonia, acute kidney failure	thrombosis of common hepatic artery	aspiration pneumonia	aspiration pneumonia, acute kidney failure	pneumonia, acute kidney failure
ICU stay, days	8	19	3	4	7	11
Postoperative day of death	18	67	3	22	11	52
Cause of death	cardiac arrest	sepsis	liver failure	respiratory failure	sepsis	sepsis

ASA, American Society of Anesthesiologists; POPF, postoperative pancreatic fistula; ICU, intensive care unit.
